# Predicting cancerlectins by the optimal *g*-gap dipeptides

**DOI:** 10.1038/srep16964

**Published:** 2015-12-09

**Authors:** Hao Lin, Wei-Xin Liu, Jiao He, Xin-Hui Liu, Hui Ding, Wei Chen

**Affiliations:** 1 Key Laboratory for Neuro-Information of Ministry of Education, Center of Bioinformatics, School of Life Science and Technology, Center for Information in Biomedicine, University of Electronic Science and Technology of China, Chengdu 610054, China; 2 School of Linguistics and Literature, University of Electronic Science and Technology of China, Chengdu 610054, China; 3 Department of Physics, School of Sciences, and Center for Genomics and Computational Biology, North China University of Science and Technology, Tangshan 063000, China

## Abstract

The cancerlectin plays a key role in the process of tumor cell differentiation. Thus, to fully understand the function of cancerlectin is significant because it sheds light on the future direction for the cancer therapy. However, the traditional wet-experimental methods were money- and time-consuming. It is highly desirable to develop an effective and efficient computational tool to identify cancerlectins. In this study, we developed a sequence-based method to discriminate between cancerlectins and non-cancerlectins. The analysis of variance (ANOVA) was used to choose the optimal feature set derived from the *g*-gap dipeptide composition. The jackknife cross-validated results showed that the proposed method achieved the accuracy of 75.19%, which is superior to other published methods. For the convenience of other researchers, an online web-server CaLecPred was established and can be freely accessed from the website http://lin.uestc.edu.cn/server/CalecPred. We believe that the **CaLecPred** is a powerful tool to study cancerlectins and to guide the related experimental validations.

Lectin is a kind of glycoprotein which can agglutinate cells^1^,^2^. Lectins can bind carbohydrate reversibly and specifically recognize diverse sugar structures but are devoid of catalytic activity. In contrast to antibodies, they are not products of an immune response. However, they can mediate a variety of biological processes e.g. host-pathogen interactions, cell-cell recognition, complement activation pathways, cell cycle regulation and apoptosis etc. As a lectin molecule, it contains typically two or more carbohydrate-combining sites. Therefore, when they react with cells, they will not only combine with the sugars on their surfaces, but also cause cross-linking of the cells and their subsequent precipitation, a phenomenon referred to as cell agglutination^3^. Lectins are found in most organisms, ranging from viruses and bacteria to plants and animals. According to the degree of their affinity with monosaccharides, these glycoproteins can be classified into five groups: mannose, galactose/N-acetylgalactosamine, N-acetylglucosamine, fucose, and sialic acid^4^. They represent a heterogeneous group of oligomeric proteins that vary widely in size, structure, molecular organization, as well as constitution of their combining sites^5^.

Different lectins differ in functions. Cancerlectin, one kind of lectins, plays a key role in the process of tumor cells interacting with each other e.g. cell adhesion, cell growth, tumor cell differentiation, metastasis and cellular infection^6^,^7^,^8^. They can be also used as a monitor when cells become cancerous, because cancerlectins can catch the instantaneous change of the glycosylated molecule, which distributes on the cells membrane, when the cell turns cancerous. In other words, cancerlectins can be the marker of tumor tissue-derived cells^9^,^10^,^11^,^12^. For example, Helix Pomatia agglutinin is an useful prognostic indicator in colorectal carcinoma^13^. Moreover, by binding to receptors on the surface of tumor cell and then causing cytotoxicity, inhibition of tumor growth or apoptosis, the cancerlectins can be used as the therapeutic of cancer therapy^14^. Galectins are a large family of cancerlectins defined by their binding specificity for β-galactoside sugars and have a broad variety of functions including mediation of cell-cell interactions, cell-matrix adhesion and transmembrane signaling^6^,^15^,^16^,^17^,^18^. Mistletoe-lectin has the function to induce the cell apoptosis and inhibit the telomerase activity^19^. Thus, the research of cancerlectins is helpful for understanding tumor development and tumor therapy. Therefore, it has been suggested that the accurate identification of the cancerlectins be very important to the discovery of tumor marker and cancer therapy. The traditional biochemical methods are an objective approach that could be used to recognize the cancerlectins. However, these methods are usually costly and time-consuming. Thus, it is desirable to develop computational methods to distinguish cancerlectins from non-cancerlectin.

To the best our knowledge, there are quite few computational methods to distinguish cancerlectins from lectins or non-cancerlectins. Damodaran *et al.*^20^ have built a database called CancerLectinDB which contain 509 cancerlectins. The database provides an easy-to-use web interface with flexibility to select an entry or a collective set of entries matching users’ criteria. Based on this database, recently, Kumar *et al.*^21^ developed a support vector machine (SVM)-based method to discriminate between cancerlectins and non-cancerlectins. An accuracy of 69.09% was obtained in 5-fold cross-validation. This method yielded quite encouraging results, and did play a role in stimulating the advancement of this area. However, further work is needed because the prediction accuracy is still far from satisfaction.

Based on the above analysis, the present study is in an attempt to improve the accuracy of cancerlectin prediction. According to a comprehensive review^22^, to establish a really useful statistical predictor for cancerlectin prediction, firstly, an objective benchmark dataset must be constructed. Subsequently, the cancerlectin samples must be formulated with an effective mathematical expression that can truly reflect their intrinsic correlation with the target to be predicted. The third step is to select a powerful machine learning method to operate the prediction by using cross-validation. Finally, a web-server should be constructed so that the model could be available to other researches.

## Results and Discussion

### Feature selection for improving accuracy

In statistical prediction, three cross-validation methods, namely independent dataset test, sub-sampling (e.g., 2, 5 or 10-fold cross-validation) test, and jackknife test are often used to evaluate the performance of the proposed methods in practical application^22^,^23^,^24^,^25^,^26^,^27^. The jackknife test always yield a unique result for a given benchmark dataset. Therefore, the jackknife test has been increasingly widely adopted by investigators to test the power of various prediction methods^28^,^29^,^30^,^31^,^32^,^33^.

According to the *g*-gap dipeptide composition in Eqs. (3, 4), for each *g* parameter, a 400-dimension vector will be produced. If we vary gap *g* from 0 to 10, we will investigate the performances of 11×400 = 4,400 feature subsets in feature selection. However, it is time-consuming when the jackknife cross-validation is used to calculate the accuracies of all feature sets. To reduce the computational time, we firstly use the 10-fold cross-validation to obtain the optimal parameters of each model. Once the optimal feature set is determined, the rigorous jackknife cross-validation will be performed to finally evaluate the anticipated success rate of the predictor.

For each gap *g*, we must find out the best feature subset which can achieve the highest accuracy. Obviously, the best feature combination can be found by examining the performance of all combinations of features. However, the computing time will be so long that it is impossible to investigate the performance of all feature sets. Taking the amino acid composition containing 20-dimension feature vector as an example, the number of all possible combinations for 20-D vector is 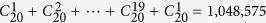. For 400 dipeptides, the number of all possible combinations will be greater than 2.58×10^120^.

In order to save computational time, we employed the ANOVA to select features in a stepwise fashion. Firstly, the difference of each feature between the two classes was measured by ANOVA *F* value as defined by Eq. 5. Hence, all features can be ranked according to their *F* values from large to small. Subsequently, the feature subset started from a feature with the highest *F* value in the ranked feature set. The SVM was employed to investigate the prediction performance of the feature subset. Thirdly, a new feature subset was produced when the feature with the second highest *F* value was added. The overall accuracy of this feature subset was also evaluated by SVM. Fourthly, this process was repeated from the higher to the lower *F* value until all candidate features were added. Then the SVM was used to examine the accuracies of all feature subsets. All examinations were performed by using jackknife cross-validation to avoid over-fitting.

Generally, the larger the feature set is, the more information the representation bears. However, the high dimension features would bring about information redundancy or noise. These would result in low capability in the generalization of a predictor or reduce the cluster-tolerant capacity so as to lower down the cross-validation accuracy. For example, the 400 1-gap dipeptides can only produce the Acc of 63.12% for discriminating between cancerlectin and non-cancerlectin. In contrast, the 1-gap dipeptides with larger *F* valve give more reliable information for classification. The occurrence of these dipeptides prefers the cancerlectins. The low dimension feature can improve the robust of a predictor. However, if 1-gap dipeptides in feature set are few, they are still not the optimal features for prediction because they cannot afford enough information and reflect real characteristics of the cancerlectins, which leads to the poor predictive accuracy. For instance, by selecting 20 1-gap dipeptides with *F* > 7.49 as input features, we can only achieve an accuracy of 68.56%.

**Figure 1:**
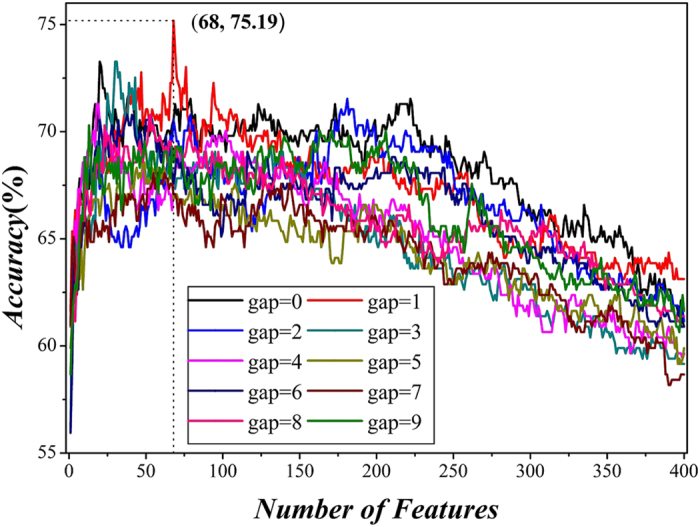
A plot to show the *g*-gap dipeptide results. When the top 68 1-gap dipeptides were used to perform prediction, the overall success rate reached its peak of 75.19%.

Therefore, the final step of feature selection is to find out the best feature subset which can produce the highest prediction accuracy. We thus plotted a curve in a 2D Cartesian coordinate system with the number of features as its abscissa and the overall accuracy as its ordinate. The maximum accuracy corresponds to the peak of the curve which can be easily observed. According to the curve shown in Fig. 1, the overall accuracy reached its peak (*Acc* = 75.19%) when the top ranked 68 1-gap dipeptides (*F* > 3.22) were used.

To compare the performance of other *g*-gap feature subsets, we repeated the process of feature selection. As we can see from Fig. 1, the feature subset with *g* = 1 and FD = 68 is the best one among the 4400 feature sets. The *Sn* and *Sp* are 69.10% and 80.10%, respectively.

### Feature analysis

**Figure 2:**
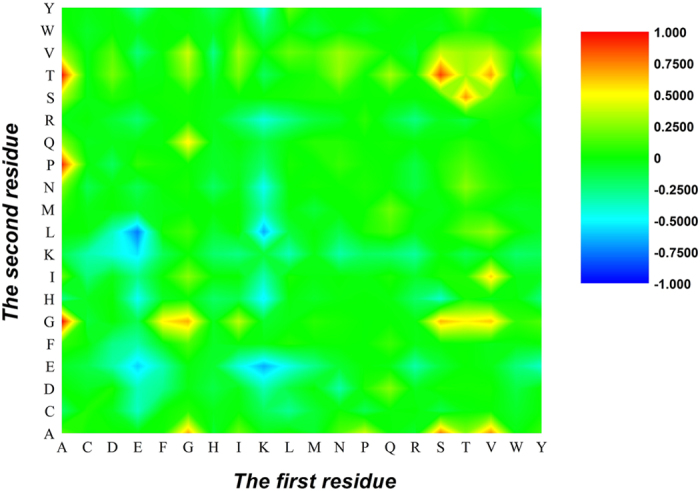
A chromaticity diagram for the *F*^0^(*u*) of 400 1-gap dipeptides. The blue boxes were positively correlated with cancerlectins, while the red boxes were negatively correlated with cancerlectins.

The results in Fig. 1 also reveal that the correlation between two residues with one residue interval (*g* = 1) is more important than other correlations in cancerlectins sequences. It is sure that some important 1-gap dipeptides contribute to the recognition of cancerlectins. We analyzed the contribution of different 1-gap dipeptides to the prediction model according to Eq.10. A heat map was shown in Fig. 2. The column and row of the heat map represent the first residue and the second residue of 1-gap dipeptides, respectively. Each element in the heat map represents a 1-gap dipeptide and is colorized according to its 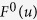. It is observed that the majority of 1-gap dipeptides have very small absolute value of 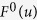 (green), indicating that these features are irrelevant with the cancerlectin prediction. We also found that the amino acids A, L, P, Q and R, (red) as well as their 1-gap correlations often appear in cancerlectins, whereas the amino acids D, G, N, T and V(blue), together with their 1-gap correlations, are not preferred in cancerlectins.

As we can see from Fig. 2, the colors of some 1-gap dipeptides are sharply different from that of other 1-gap dipeptides. We cautiously picked out 21 1-gap dipeptides (L*R, P*A, N*D, N*V, Q*P, Q*L, N*W, D*T, N*G, R*R, A*P, T*H, H*M, L*E, K*M, P*H, L*P, T*D, Q*A, P*Q, R*Q) according to the criteria that the absolute value of 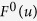 is larger than 0.5. Among the 21 features, 15 features in Fig. 2 are marked in red, indicating that the occurrence frequencies of these features in cancerlectins are dramatically larger than that in non-virion proteins. Only 6 1-gap dipeptides in Fig. 2 marked in blue prefer non-cancerlectins. The reason of this phenomenon is that non-cancerlectin dataset is consisted of other lectins. The features of different lectins are annihilated each other. Thus, according to the strategy in the outer membrane protein and promoter prediction^34^,^35^, it is better to use multi negative sets, in which each negative set has its given type, to train and test the model. However, in this study, the currently available data do not support the strategy. Otherwise, the proteins for some subsets are too few to be statistically significant. However, these 21 features do play important roles in cancerlectin prediction and yield the Acc of 68.32% in 5-fold cross-validation, dictating that the ANOVA-based feature selection technique is powerful.

### Comparison with other methods

A comparison is made between the proposed method and other published methods. The comparative results of different methods on the same benchmark dataset are listed in Table 1. Kumar *et al.*^21^ has investigated the accuracies of the split based composition (2-part and 4-part), Position-Specific Scoring Matrix (PSSM) and PSSM combined with PROSITE domains by using SVM. They found that PSSM combined with PROSITE domains can achieve the highest accuracy of 69.09%. However, the accuracy of our proposed method is even higher than that of Kumar *et al.*^21^, demonstrating that our method is a more powerful method in identifying cancerlectins. Moreover, the PSSM information also has shortcomings. The generation of PSSM of a protein depends largely on the searching dataset. If no homologous sequence is found in the searching dataset, the PSSM will not give exact description, thus leading to wrong prediction. With primary sequence information, our model can obtain such high accuracy, suggesting that the proposed model is more neat free and efficient.

Furthermore, we also investigated the *Acc* achieved by completely random guess (CRG). Obviously, the *Acc* achieved by CRG is 50.00%. If considering the weight or prior probability, the *Acc* is [178×(178/404) + 226×(226/404)]/404 = 50.71%. These results demonstrate that our method is superior to the published methods and random guess.

Moreover, using ANOVA to perform feature selection has many advantages as follows. Firstly, it is robust to most violations of its assumptions. Secondly, it is more intuitive for user to analyze the interaction of the two variables. Thirdly, it can be used effectively even when the number of observations is different in each group. Finally, it can be easily generalized to more than two groups without increasing the Type I error. By using ANOVA to select features, the important features were picked out, which improve the cross-validated accuracies and robust of model. Thus, it is reasonable that our method has better performance.

It is well known that the physiochemical properties of amino acids and their correlation play important roles in protein structure and function. Thus, in the future, we will make our effort to study their roles in cancerlectins. We hope that the accuracy will be improved by combining the *g*-gap dipeptide composition with physiochemical properties of amino acids.

### Web-Server Guide

Establishing a user-friendly web-server will improve the efficiency and avoid repeating a complicated mathematics and program for studying cancerlectins. The predictor established via aforementioned procedures is called **CaLecPred**. For the convenience of the vast majority of experimental scientists, we provided a guide to help experimental scientists to use the web-server to get the desired results.

**Figure 3:**
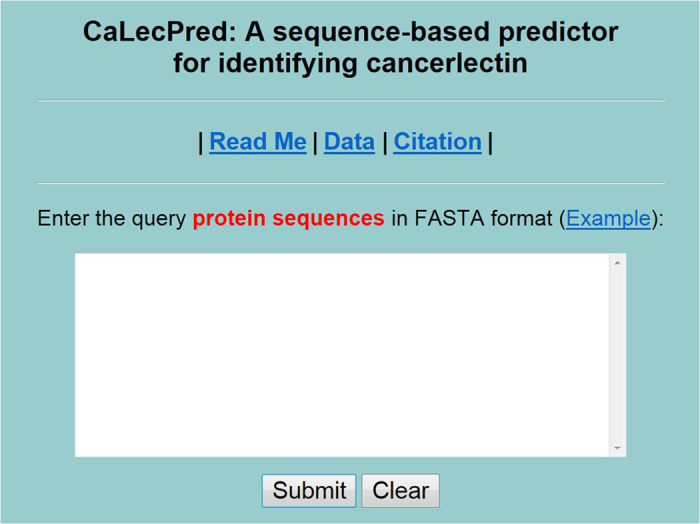
A semi-screenshot to show the top page of the CaLecPred webserver. Its website address is http://lin.uestc.edu.cn/server/CaLecPred.

Firstly, browse the web server at http://lin.uestc.edu.cn/server/CaLecPred and you will see the top page of **CaLecPred** on your computer screen, as shown in Fig. 3. Click on the Read Me button to see a brief introduction about the predictor and the caveat when using it. Click on the Data button to download the benchmark datasets used to train and test the **CaLecPred** predictor. Click on the Citation button to find the relevant papers that document the detailed development and algorithm of **CaLecPred**. Secondly, either type or copy/paste the query lectin sequences into the input box at the center of Fig. 3. The input sequence should be in the FASTA format. Example sequences in FASTA format can be seen by clicking on the Example button right above the input box. Thirdly, click on the Submit button to see the predicted result. It should be noted that each of the input query sequences should exclude all illegal characters: such as ‘B’, ‘X’, ‘U’, ‘Z’.

To further examine the performance of the web-server, a total of 40 lectins including 20 cancerlectins and 20 non-cancerlectins were collected manually from Uniprot and NCBI. These lectins are independent from training dataset and can also be downloaded from http://lin.uestc.edu.cn/server/CaLec/data.html. Then we used the independent data to compare the performance between **CaLecPred** and the previously published web-server called CancerPred^21^. Because the servers based on PSSM and PROSITE-PSSM of CancerPred are not available, we only examined the accuracies of CancerPred based on amino acid composition, dipeptide composition, split composition (2-part), and split composition (4-part). Comparative results were listed in Table 2. As shown in the table, **CaLecPred** can achieve the maximum overall accuracy, demonstrating that **CaLecPred** is superior to CancerPred.

Although the proposed method achieved encouraged results and has been applied in other bioinformatics fields^31^,^36^,^37^, more studies are still needed to validate our findings for generalization of our method.

## Conclusions

In this paper, we developed a novel approach for the prediction of cancerlectins. In order to improve the prediction capability of model, the ANOVA-based feature selection technique was utilized to optimize *g*-gap dipeptide compositions. An overall accuracy of 75.19% was achieved. By comparing with the other existing methods, we demonstrated that our proposed method is superior to other methods, suggesting that **CaLecPred** is a powerful tool for the study in discriminating between cancerlectin and non-cancerlectin.

## Material and Method

### Dataset

A reliable and objective benchmark dataset is a key point in building a power classifier. The original dataset was obtained from Kumar *et al.*^21^ who extracted the protein annotation information and sequences from **CancerlectinDB** at http://proline.physics.iisc.ernet.in/cgi-bin/cancerdb/input.cgi^20^. After removing duplicated sequences and sequences without experimental evidence, or containing non-standard amino acids, 385 proteins were obtained to form the positive dataset. A negative dataset including 820 proteins was built by searching the **UniProt** Database (http://www.uniprot.org/) using the keyword “lectin” and then removing sequences tagged with “similar”, “fragment”, “putative” and “probable”.

Generally, if a designed dataset contains highly similar sequences, misleading results with overestimated accuracies will be obtained and the generalization ability of the proposed model will be reduced^38^. To remove the homologous sequences from the benchmark dataset, a cutoff threshold of 25% was recommended to exclude those protein/peptide sequences from the benchmark datasets that had ≥ 25% pairwise sequence identity to any other sample in the same subset^39^,^40^. However, in this study we did not use such a stringent criterion because the currently available data did not allow us to do so. Otherwise, the peptides would be too few to be statistically significant. Thus, the CD-HIT program^41^ was employed with 50% as the sequence identity cutoff to remove redundant sequences. As a result, in total, 178 cancerlectin and 226 non-cancerlectin sequences were obtained and can be formulated as follows:


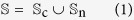


where the subset 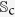 contains 178 cancerlectin samples, 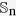 contains 226 non-cancerlectin samples, while the symbol 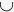 represents the union in the set theory.

### The representation of sequence samples

Given a protein **P** with L amino acids, how to translate it into a mathematical expression for statistical prediction is the first major concern to develop a sequence-based predictor for identifying cancerlectins. The most straightforward method to formulate the sample of a protein **P** with L residues is to use its entire amino acid sequence, which can be formulated by


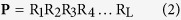


where R_1_ represents the 1st residue of the proteins, R_2_ the 2nd residue of the protein, and so forth. Subsequently, we can utilize various sequence-similarity-search-based tools, such as BLAST, to perform statistical prediction. Although this kind of sequence model was very straightforward and intuitive, unfortunately it failed to work when a query protein did not have significant similarity to any of the protein sequences in the training dataset. Thus, investigators turned to use vectors to represent the peptide samples. Another reason for them to do so is that the statistical samples in vector format are much easier to be handled than in sequence format by many existing operation engines.

Another common strategy is to formulate protein sequences with amino acid composition (AAC)^42^. To obtain the sequence-order information, the simple AAC was replaced by the adjoining dipeptide composition to represent the sample of a protein^37^,^43^. However, the adjoining dipeptide composition can only reflect the short-range correlation. Generally, the intrinsic properties of protein sequences may be deposited in higher tier correlation of residues^44^,^45^. The interval residues in primary sequence are spatially closer in tertiary structure which means that interval residues is more significant than the adjacent residues in biology. Especially, in some regular secondary structures, such as alpha helix and beta sheet, two non-adjoining residues are connected by hydrogen bonds. Thus, to search for the important correlation, we extended the adjacent dipeptide composition to the *g*-gap dipeptide composition^46^ which can be used to describe the correlation between two residues with *g* residues.

Thus protein **P** can be formulated by


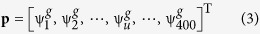


where the 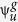 is the frequency of the *u*-th (*u* = 1, 2, …, 400) *g*-gap dipeptide and calculated by


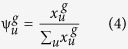


here 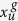 denote the number of the *u*-th *g*-gap dipeptide in a protein. Note that when *g* = 0, the *g*-gap dipeptide will degenerate to the adjoining dipeptide composition.

### Feature Selection

It has been proved that the optimized parameters could improve predictive accuracy^47^,^48^,^49^,^50^. Moreover, the high dimension vector in feature set would cause dimension disaster and will lead to a handicap for the computation or increase of computational time^38^. Thus, a wise strategy is to use feature selection techniques to find the optimal feature set, which will not only gain deeper insights into the intrinsic properties of protein sequences, but economizing runtime and computational resource. Currently, some methods like principal component analysis, genetic algorithm and minimal redundancy maximal relevance have been presented for feature selection^51^,^52^. A statistics-based algorithm, called the analysis of variance (ANOVA), has been proposed to rank the important of features and yielded good results^36^,^45^,^46^. Thus, ANOVA-based feature selection technique was also used here to find out the best feature set which can achieve the maximum accuracy.

The principle of ANOVA is to calculate the ratio (*F* value) of features between groups and within groups for measuring feature variances. Then the *F* value (*F*(*u*)) of the *u*-th feature in benchmark dataset is defined by:


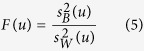


where 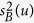 and 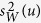 are the sample variance between groups (also called Means Square Between, MSB) and sample variance within groups (also called Mean Square Within, MSW), respectively. They are given by:


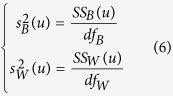


here *df*_*B*_ = *K*−1 and *df*_*W*_ = *N*-*K* are degrees of freedom for MSB and MSW, respectively. *K* and *N* represent the number of groups (here *K* = 2) and total number of samples (here *N* = 404), respectively. *SS*_*B*_(*u*) and *SS*_*W*_(*u*) are sum of squares between groups and sum of squares within groups, respectively, which can be calculated by


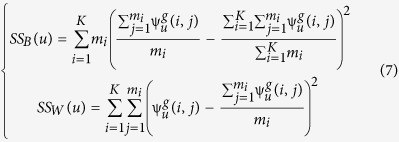


where 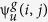 denotes the frequency of the *u*-th *g*-gap dipeptide of the *j*-th sample in the *i*-th group; *m*_*i*_ denotes the number of samples in the *i*-th group (here *m*_1_ = 178, *m*_2_ = 226).

Obviously, a large value of *F*(*u*) means that the *u*-th feature has a better discriminative capability. Hence, all features can be ranked according to their *F* values. Subsequently, the incremental feature selection (IFS)^46^,^53^ was used to determine the optimal number of features as described below. Firstly, the feature subset started from a feature with the highest *F* value in the ranked feature set. Secondly, a new feature subset was produced when the feature with the second highest *F* value was added. This process was repeated from the higher *F* to the lower *F* value until all candidate features were added. Thus, for any gap *g*, the 400 feature subsets will be produced. The *ε*-th feature subset is composed of *ε* ranked *g*-gap dipeptides and can be expressed as:


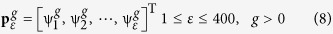


For each of the 400 feature sets, the prediction accuracy of the proposed method was examined on the benchmark dataset by using jackknife cross-validation. Then we obtained an IFS curve in a 2D Cartesian coordinate system with index *ε* (the number of features) as its abscissa (or *X*-coordinate) and the overall accuracy as its ordinate (or *Y*-coordinate). If *g* varies from 0 to *g*_*θ*_, there are *g*_*θ*_ + 1 IFS curves. The peak (the maximum accuracy) can be observed in these curves. Then the optimal feature subset with parameters *ε*_0_ and *g*_*ϕ*_ can be determined and expressed as:


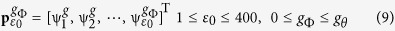


where *ε*_0_ is the number of optimal 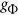-gap dipeptides.

Based on above processes, the high-dimensional data will be projected into a low-dimensional space. The final classifier model was built based on the optimal feature subset.

### Feature analysis

To provide an overall and intuitive view, the following normalized function was introduced to scale the *F*(*u*) of the *u*-th *g*-gap dipeptide as follows


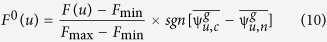


where *F*_min_ and *F*_max_ are the minimum and maximum *F* values of all the 400 *g*-gap dipeptides. The 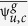 and 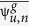 are the average frequencies of the *u*-th *g*-gap dipeptide in cancerlectins and non-cancerlectins, respectively; sgn is the sign function. Thus, we have 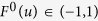. If 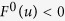, the *u*-th *g*-gap dipeptide prefers cancerlectins, otherwise it prefers phage non-cancerlectins.

### Support vector machine

Support Vector Machine (SVM) is a kind of learning machine method based on statistical learning theory and has been widely used in the field of bioinformatics^54^,^55^,^56^,^57^,^58^,^59^,^60^,^61^. The basic idea of applying SVMs to pattern classification can be summarized as follows. In this study, the software LibSVM designed by Lin’s lab was used to implement SVM. Empirical studies have demonstrated that the radial basis function (RBF) outperforms the other three kinds of kernel functions (linear function, polynomial function, sigmoid function) in classification^62^,^63^. Thus the RBF kernel function was used in the current work. A grid search method was used to optimize the regularization parameter *c* and kernel parameter *γ* by using cross-validation test. The search spaces for *c* and *γ* are [2^15^, 2^−5^] and [2^−5^, 2^−15^] with steps being 2^−1^ and 2, respectively.

### Performance assessment

To provide a simple method to measure the prediction quality, the following three metrics: sensitivity (*Sn*), specificity (*Sp*) and accuracy (*Acc*) were used and expressed as


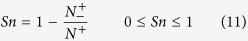



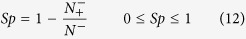



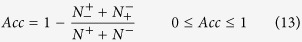


where *N*^+^ and *N*^−^ denote the number of cancerlectins and the number of non-cancerlectins, respectively; 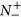 and 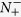 are the number of the cancerlectins incorrectly predicted as the non-cancerlectins and the number of the non-cancerlectins incorrectly predicted as the cancerlectins, respectively.

## Additional Information

**How to cite this article**: Lin, H. *et al.* Predicting cancerlectins by the optimal *g*-gap dipeptides. *Sci. Rep.* **5**, 16964; doi: 10.1038/srep16964 (2015).

## Figures and Tables

**Table 1:** Comparison with other published methods on training data.

Methods	*Sn* (%)	*Sp* (%)	*Acc* (%)
Split based composition(2-part)^21^	66.32	64.18	65.10
Split based composition(4-part)^21^	65.12	66.85	66.09
PSSM^21^	67.92	68.57	68.34
PSSM with 14 PROSITE domains^21^	68.00	69.90	69.09
Our method	69.10	80.10	75.19

**Table 2:** Comparison with other web server on independent data.

Web server	*Sn* (%)	*Sp* (%)	*Acc* (%)
CancerPred (Amino acid composition)^21^	90.00	55.00	72.50
CancerPred (Dipeptide composition)^21^	70.00	65.00	67.50
CancerPred (Split composition (2-part))^21^	85.00	75.00	80.00
CancerPred (Split composition (4-part))^21^	70.00	95.00	82.50
**CaLecPred**	80.00	90.00	85.00
